# 5-HT1A and 5-HT2A Signaling, Desensitization, and Downregulation: Serotonergic Dysfunction and Abnormal Receptor Density in Schizophrenia and the Prodrome

**DOI:** 10.7759/cureus.15811

**Published:** 2021-06-21

**Authors:** Sun A Kim

**Affiliations:** 1 General Surgery, University of Central Florida College of Medicine, Orlando, USA

**Keywords:** prodrome, schizophrenia and other psychotic disorders, selective serotonin reuptake inhibitor, receptor desensitization and downregulation, 5-hydroxytryptamine

## Abstract

The significant role of serotonin (5-hydroxytryptamine [5-HT]) in the pathogenesis and early development of schizophrenia has been established by contemporary research through the assessment of structural and pharmacological neuroimaging, blood metabolites, cerebrospinal fluid, genome polymorphisms, and other valid indicators of abnormal serotonergic activity in prodromal, ultra-high-risk, and schizophrenic patient groups. A modern approach toward understanding the complex psychophysiology behind schizophrenia will be outlined through the demonstration of 5-HT_1A_ and 5-HT_2A_ receptors as key modulators within the spectrum of negative symptoms associated with schizoaffective disorders, including a variety of disturbances in cognition, behavior, mood, social function, perception of reality, and hormonal response to stressors. This paper will review the evidence for attributing the risk of schizophrenia onset to early defects in serotonergic neurotransmission and explore the perspective of selective serotonin receptor inhibitor (SSRI) pharmacotherapy as a method of treatment and intervention for prodromal and ultra-high-risk patients by increasing 5-HT_1A _receptor sensitivity levels and modifying the transcription of 5-HT_1A_ receptor-associated gene expression in these groups.

## Editorial

Introduction

For several decades, dopamine (DA) has been considered the most important neurotransmitter in the physiology and etiology of schizophrenia due to the blocking effect demonstrated by all traditional or “typical” antipsychotics upon the dopamine D2 receptor. Although the dopamine hypothesis of schizophrenia still remains the primary explanation behind the physiological basis and mechanism of action in schizophrenia today, it has been increasingly demonstrated over time that a number of additional neurotransmitter systems, including serotonin (5-HT), glutamate, gamma-aminobutyric acid (GABA), opioids, and trace amines, are involved in the development of the disorder. 5-HT_1A_ and 5-HT_2A_, which are among the seven families of 5-HT receptors, are known to play a significant role in the treatment of patients with various mood and/or psychotic disorders. Recent evidence for the naturally occurring upregulation of 5-HT_1A_ and downregulation of 5-HT_2A_ receptors throughout the lifetime of schizophrenic patients supports the role of serotonin as a primary factor in the developmental delay and discourse of schizophrenia. New intervention strategies involving the functional relationship between 5-HT_1A_ and 5-HT_2A_ receptors may be employed in the future to prevent, mitigate, or delay the onset of positive and negative symptoms associated with full-blown schizophrenia before and after the first episode of psychosis.

Pathogenesis and neurodevelopment

Evidence supporting the early predisposition for schizophrenia consists of a variety of structural changes, including ventricular distension, increase in gliosis, reduced cortical folding, and loss of general brain tissue. Furthermore, magnetic resonance imaging (MRI) studies have found that compared with controls, patients with schizophrenia show significantly reduced folding in the anterior cingulate cortex (ACC) that differs from conventional left ACC sulcal symmetry. Given that sulcal/gyral folding is almost complete by the third trimester of gestation and remains relatively stable shortly after birth, the structural anomalies found in ACC folding are likely to reflect early prenatal neurodevelopmental factors in the etiology of schizophrenia [1].

Structural neuroimaging studies have revealed the limited progression of changes in the brain during the prodromal, transitional, and onset stages of schizophrenia, thereby supporting the notion that schizophrenia is a neurodevelopment disorder that may be characterized by early structural and physiological abnormalities within the brain. Researchers have investigated brain structure in large numbers of young people at-risk (AR) for the development of psychosis using MRI. Stone et al. (2011) revealed that subjects at-risk for psychosis show significantly reduced activation in the left parahippocampal gyrus during an episodic encoding task through fMRI and proton magnetic resonance spectroscopy (^1^H-MRS) [2]. A separate comparison study between baseline and 12-month follow-up scans for patients at-risk for psychosis indicates a reduction of gray matter in the left cerebellum. It is unsurprising that the high-risk population had smaller volumes of the left hippocampus in comparison to healthy controls; however, retrograde survival analyses have yielded unexpected results: the lack of change in left hippocampal volume was associated with a future transition to psychosis, and a significantly reduced left hippocampal volume was only seen in patients who not develop acute psychotic symptoms [2]. These results imply that abnormalities in hippocampal volume and transmission, among other structural defects, precede the clinical expression of psychosis and may serve as predictive indicators for conversion to schizophrenia.

The ability to characterize premorbid neurostructural changes with the neurodevelopment of schizophrenia or lack thereof supports the notion that prodromal, at-risk (AR), or ultra-high-risk (UHR) groups may greatly benefit from early assessment and intervention strategies prior to the first episode of psychosis.

Serotonin (5-HT)

**Figure 1:**
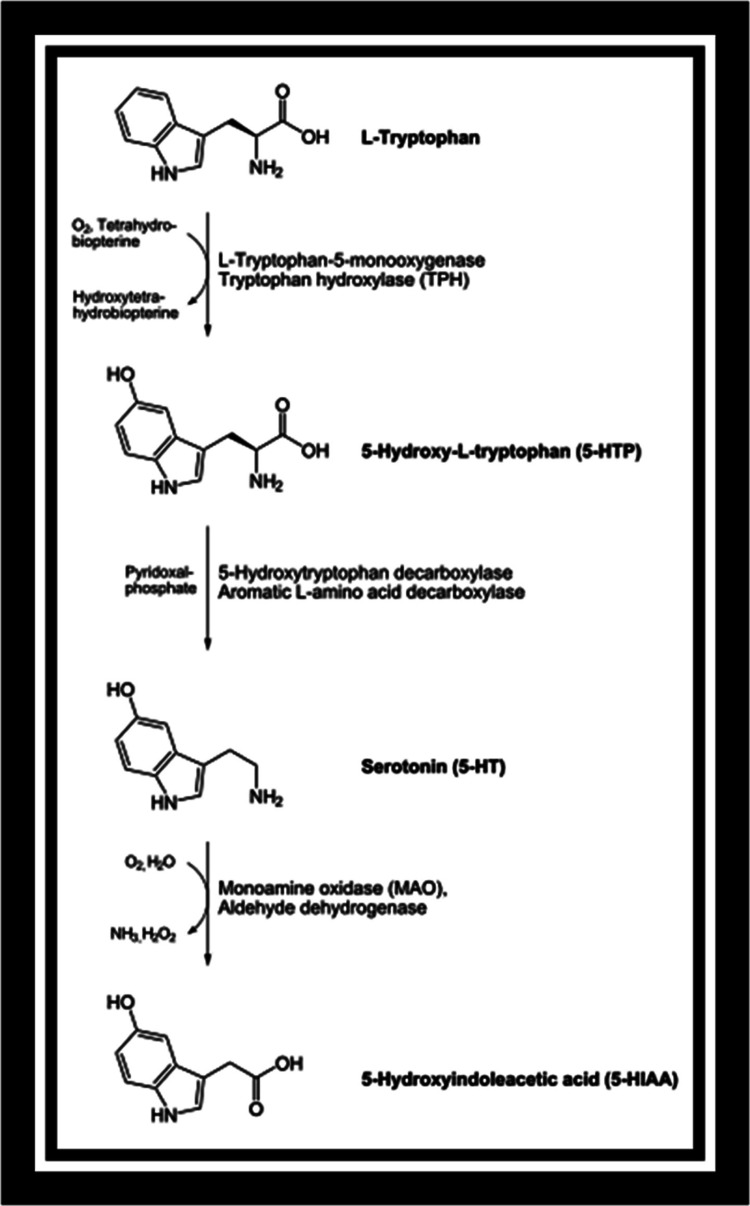
Biosynthesis of serotonin Increased aromatic amino acid decarboxylase (AADC) and decreased monoamine oxidase (MAO) activity have been observed in schizophrenic patients. Both of these enzymes are expected to heavily influence levels of serotonin in the bloodstream.

Figure 1 shows the biosynthesis of serotonin.

5-HT_1A_R and 5-HT_2A_R in psychosis and schizophrenia

There is a great deal of evidence that demonstrates the 5-HT system as the primary regulator of dopaminergic activity in the brain (and vice versa). Of the 14 different subtypes of serotonin receptors in the brain, the 5-HT_1A_ and 5-HT_2A_ receptors currently hold the leading role in the pathophysiology of schizophrenia. 5-HT_1A_ and 5-HT_2A_ receptors are frequently co-localized on the same cell, although they are known to elicit oppositional responses. An imbalanced 5-HT_1A_ and 5-HT_2A_ receptor ratio is considered a significant causal factor during the early development of abnormalities in the schizophrenic brain. Furthermore, abnormal receptor density ratios are strongly associated with positive and negative symptom severity, which are typically assessed using the Scale for the Assessment of Negative Symptoms (SANS) and the Scale for the Assessment of Positive Symptoms (SAPS).

**Figure 2:**
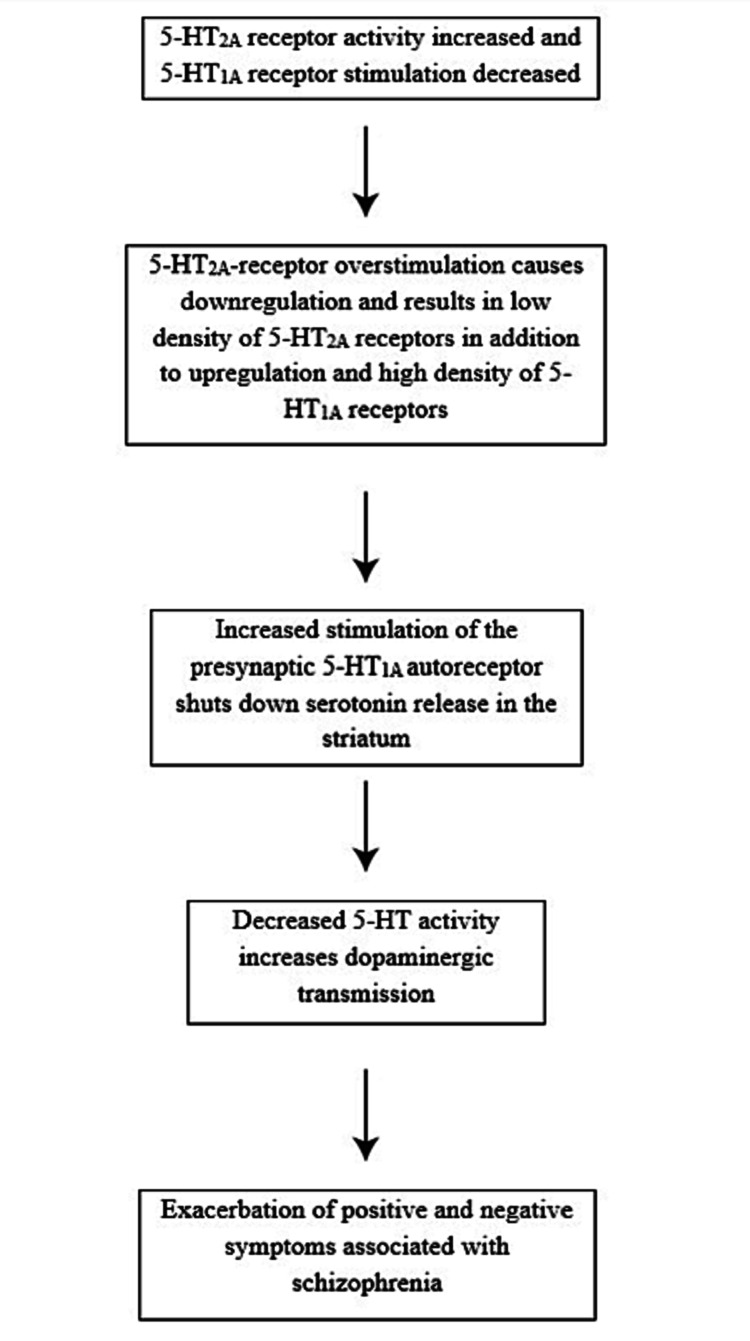
Pathogenesis of schizophrenia as it relates to serotonergic neurotransmission This figure serves as a model for understanding the development of schizophrenia with emphasis placed on the upregulation of 5-HT_1A_ and downregulation of 5-HT_2A_ receptor subtypes.

Postmortem studies have varying claims - some have argued that patients with schizophrenia have significantly different receptor ratios in comparison to healthy controls while others have maintained that there is no statistically significant difference between the two groups. Several positron emission tomography (PET) studies using various ligands (e.g. ^[18F]^Setoperone) to examine neuroleptic-naive schizophrenic patients in-vivo have shown increased 5-HT_1A_ and decreased 5-HT_2A_ receptor densities primarily in the dorsolateral prefrontal cortex, the region of the brain that is most associated with both positive and negative symptoms [3]. In addition, Hurlemann et al. (2007) proposed that 5-HT_2A_ receptor density was also decreased in the At-Risk Mental State patient subgroup, regardless of conversion to psychosis [4]. The progressive decline in subcortical 5-HT_2A_ receptor density could provide an indicator of conversion to schizophrenia. The enhancement of 5-HT_1A_ receptor density in several regions of the brain associated with schizophrenia may be caused by inadequate stimulation of these receptors due to the overstimulation of 5-HT_2A_ receptors, as shown by the decrease in 5-HT_2A_R to accommodate such intense dopaminergic neurotransmission (Figure 2).

Drug models for understanding 5-HT

The earliest hypotheses regarding the involvement of 5-HT in schizophrenia were developed based on the psychotomimetic and hallucinogenic effects of lysergic acid diethylamide (LSD), as well as the reversal role that is played by 5-HT receptor antagonists in the brain. Although evidence supports the notion that 5-HT_2A_ receptors have significant interactions during psychosis elicited by drugs such as LSD and dimethyltryptamine (DMT), the effects of chemicals on the mental state is still unclear to this day. Strassman et al. (1996) showed that 5-HT_1A_ suppresses DMT’s hallucinogenic activity through conducting a study that involved the co-administration of pindodol, a 5-HT_1A_ antagonist, with a sub-hallucinogenic dose of DMT (0.1 mg/kg IV) [5]. The result showed a three-fold enhancement of DMT’s psychopharmacological effects, thereby indicating that the 5-HT_1A_ receptor is, indeed, a major regulator of neurochemical response to hallucinogenic drugs.

In 1997, Simpson et al. suggested that 5-HT_1A_ modulation of glutamatergic activity might be abnormal in schizophrenia due to the fact that some 5-HT_1A_ receptors are located on pre or post-synaptic elements of glutamatergic synapses found in schizophrenic patients [6]. In comparison to dopaminergic agonists, such as amphetamine, or serotonergic agonists, such as LSD and DMT, non-competitive N-methyl-D-aspartate receptor (NMDAR) antagonists, such as phencyclidine (PCP), ketamine, and MK-801, are considered a more faithful method of reproducing psychosis and the full spectrum of cognitive deficits and psychopathological symptoms characterized by schizophrenia.

5-HT plays a significant role in attenuating or exacerbating a wide range of behavioral and biochemical effects of NMDAR non-competitive antagonists in rodent and primate models used to understand the mechanisms of schizophrenia and of atypical antipsychotic drugs [7]. The mitigation of hyperlocomotor effects through the use of selective 5-HT_2A/2C_ NMDAR inverse agonists, which are much more potent at 5-HT_2A_ than 5-HT_2C_ receptors, strongly supports the leading role of 5-HT_2A_ in the NMDAR antagonist model of psychosis [7].

Pharmacotherapy

Treatment of schizophrenia using the classical antipsychotic drugs, all of which are potent D2 receptor antagonists, has also been shown to induce hyperprolactinemia, which may lead to serious health problems, including sexual dysfunction, osteoporosis, and amenorrhea. Recently, new pharmacological strategies have been employed in the treatment of schizophrenia to alleviate the psychotic symptoms associated with abnormal dopamine (DA) activity and reduce the extrapyramidal side (EPS) effects (tardive dyskinesia, dystonia, pseudoparkinsonism, and akathisia) caused by classical antipsychotics, which are primarily D_2_ receptor antagonists. EPS is known to occur when approximately 80% of striatal D_2_ receptors are occupied in the nigrostriatal pathway, which is involved with motor control. Classical antipsychotics also serve as D_2_ blockers in the mesolimbic, mesocortical, and tuberoinfundibular pathways, which are considered to be respectively associated with the positive symptoms of schizophrenia, cognitive deficits, and control of pituitary prolactin secretion [8].

Newer “atypical” psychotics primarily serve as antagonists at the 5-HT_2A _and D_2_ receptors; however, they also select for a cohort of other receptors with varying binding affinities. Atypical antipsychotics, such as clozapine, which possesses the highest affinity ratio for 5-HT_2A_ relative to D_2_ receptors as assessed in the Clinical Antipsychotic Trials of Intervention Effectiveness (CATIE) studies, have been proven to achieve efficacy with significantly less EPS than classic D_2_ receptor antagonists in the treatment of schizophrenia [9]. Though it is clear that the regulation of nigrostriatal dopaminergic transmission through the 5-HT_2A_ receptor is effective in the reduction of EPS, both laboratory research and clinical observations indicate that 5-HT_2A_ receptor antagonism is unable to provide full protection. The 5HT_1A_ receptor subset is a potential target for the treatment of schizophrenia because it is known to be involved in the mitigation of anxiety and depression, protection from EPS, and counteractive to the side effect of weight gain caused by typical and atypical antipsychotics [9]. Although several atypical antipsychotics exhibit partial 5-HT_1A_ receptor agonist activity, we still have much to learn about its role in the treatment of schizophrenia.

5-HT-mediated behaviors are considered important pharmacological tools when examining the efficacy of pharmacotherapy on psychopathology. The continuous administration of precursors of 5-HT such as tryptophan and 5-HTP at high doses with a monoamine oxidase inhibitor generally enhances positive symptoms in schizophrenic patients. The displacement of endogenous DA resulting from high doses of 5-HT formed from precursors is most likely the method of this positive effect [10]. Early studies in the development of ACP-103, a selective 5-HT_2A_ inverse agonist that is devoid of D2 binding, demonstrates a pharmacologic profile comparable to that of an atypical antipsychotic, for it “1) Inhibits head-twitching behavior elicited by the 5-HT_2A/2C_ agonist (+)-2,5-dimethoxy-4-iodoamphetamine (DOI), 2) attenuates hypolocomotor activity produced by the NMDA receptor antagonist MK-801, and 3) restores DOI-induced disruption of prepulse inhibition” [11] without the impairment of cognition or development of catalepsy. Although it has also been shown that ACP-103 does not reverse hyperactivity induced by amphetamine when administered alone, co-administration with typical antipsychotics as an adjunctive therapy results in a synergistic effect that blocks amphetamine and MK-80-induced hyperactivity without increasing propensity for catalepsy and hyperprolactinemia [9].

The contemporary hypothesis regarding the mechanism of antidepressant drug action is that the enhancement of serotonergic neurotransmission is largely due to transcriptional gene regulation and cellular signaling of 5-HT_1A_ and 5-HT_2A_ receptors. The continuous activation of 5-HT_2A_ receptors has been found to increase the sensitivity level of postsynaptic 5-HT_1A_ receptors and modify the transcriptional expression of the 5-HT autoreceptor [12]. Currently, mood disorders (i.e. depression and anxiety) have been treated through the administration of selective serotonin reuptake inhibitors (SSRIs) such as sertraline or fluoxetine. Chronic administration of an SSRI is expected to increase serotonin levels in the brain by desensitization of the 5-HT_1A_ autoreceptor and inhibition of the uptake of serotonin, thereby inducing a more pronounced release of serotonin. This may prove to be a more effective form of antidepressant therapy in depressed patients that do not respond as well as others to antidepressant drugs, as well as patients whose 5-HT_1A_ receptors have become desensitized either through uptake blockers or prolonged administration of 5-HT agonists. The vast spectrum of responses that have been generated by SSRI treatment suggests that 5-HT receptor sensitivity levels largely vary, irrespective of psychotic symptoms.

The exacerbation of positive symptoms is associated with the increased activity of mesolimbic DA neurons. Deficits in dopaminergic activity in the nigrostriatal and mesocortical systems correlate to negative symptoms and EPS [13]. It is likely that antagonism at 5-HT_2_ receptors contributes to the beneficial effects of some drugs in the amelioration of positive symptoms associated with schizophrenia. However, studies using the atypical antipsychotic amisulpride show that 5-HT_2_ receptor antagonism is not essential for the reduction of certain negative symptoms, which remain untreated as shown by score subsets on diagnostic measures such as the Positive and Negative Syndrome Scale (PANSS), Global Assessment of Functioning (GAF), and Brief Psychiatric Rating Scale (BPRS). In particular, typical and atypical antipsychotic medications have been shown to be clinically effective at ameliorating anxiety but ineffective at improving negative symptoms such as social withdrawal, anhedonia, avolition, blunted affect, and poverty of thought and speech content [8].

It is known that a mixed effect takes place once 5-HT_1A_ receptors change in density - a reduction in post-synaptic 5-HT_1A_ receptors is involved in anxiety while higher levels of presynaptic 5-HT_1A_ receptors inhibit serotonin neurotransmission and can lead to behavioral symptoms of depression [14]. In a study involving chronic, treatment-resistant schizophrenic patients, Goff et al. (1995) found that the addition of fluoxetine to typical antipsychotics caused significant improvements specifically of negative symptoms [15]. Therefore, the use of an antidepressant that preferentially desensitizes 5-HT_1A_ autoreceptors and increases the synaptic transmission of serotonin may have a higher potential as an adjunctive treatment for targeting the negative symptoms that remain present for most patients with schizophrenia.

Conclusion

The model of 5-HT as a significant guide for assessing prodromal predisposition to schizophrenia suggests that pharmacotherapy using drugs that correct or restore disrupted 5-HT pathways and abnormal receptor densities may be useful for the treatment of positive and negative symptoms in prodromal and schizophrenic patients. Recently, early intervention approaches using atypical antipsychotics and/or cognitive behavioral therapy have shown promising potential for decreasing the duration of psychosis and improving short-term clinical outcomes. Although a pharmacogenetic test response to clozapine has been proposed to predict response to treatment with antipsychotic drugs [16], other methods of pharmacogenetic testing involving the 5-HT pathway should be developed to predict response to treatment and likelihood for developing schizophrenia in prodromal and ultra-high-risk patients. Such testing may help guide early intervention strategies towards the restoration or correction of the most vulnerable brain regions involved during different stages of the illness. Realistically, long-term studies that use a large sample of prodromal and ultra-high-risk patients with a serotonergic component of vulnerability for developing schizophrenia are necessary to accurately characterize the benefits and potential risks associated with this phase-specific psychopharmacological intervention.
